# Microbial mineral colonization across a subsurface redox transition zone

**DOI:** 10.3389/fmicb.2015.00858

**Published:** 2015-08-28

**Authors:** Brandon J. Converse, James P. McKinley, Charles T. Resch, Eric E. Roden

**Affiliations:** ^1^Department of Geoscience, University of Wisconsin-MadisonMadison, WI, USA; ^2^Pacific Northwest National Laboratory, RichmondWA, USA

**Keywords:** subsurface sediments, redox transition, minerals, colonization, amplicon sequencing

## Abstract

This study employed 16S rRNA gene amplicon pyrosequencing to examine the hypothesis that chemolithotrophic Fe(II)-oxidizing bacteria (FeOB) would preferentially colonize the Fe(II)-bearing mineral biotite compared to quartz sand when the minerals were incubated *in situ* within a subsurface redox transition zone (RTZ) at the Hanford 300 Area site in Richland, WA, USA. The work was motivated by the recently documented presence of neutral-pH chemolithotrophic FeOB capable of oxidizing structural Fe(II) in primary silicate and secondary phyllosilicate minerals in 300 Area sediments and groundwater (Benzine et al., 2013). Sterilized portions of sand+biotite or sand alone were incubated *in situ* for 5 months within a multilevel sampling (MLS) apparatus that spanned a ca. 2-m interval across the RTZ in two separate groundwater wells. Parallel MLS measurements of aqueous geochemical species were performed prior to deployment of the minerals. Contrary to expectations, the 16S rRNA gene libraries showed no significant difference in microbial communities that colonized the sand+biotite vs. sand-only deployments. Both mineral-associated and groundwater communities were dominated by heterotrophic taxa, with organisms from the *Pseudomonadaceae* accounting for up to 70% of all reads from the colonized minerals. These results are consistent with previous results indicating the capacity for heterotrophic metabolism (including anaerobic metabolism below the RTZ) as well as the predominance of heterotrophic taxa within 300 Area sediments and groundwater. Although heterotrophic organisms clearly dominated the colonized minerals, several putative lithotrophic (NH_4_^+^, H_2_, Fe(II), and HS^-^ oxidizing) taxa were detected in significant abundance above and within the RTZ. Such organisms may play a role in the coupling of anaerobic microbial metabolism to oxidative pathways with attendant impacts on elemental cycling and redox-sensitive contaminant behavior in the vicinity of the RTZ.

## Introduction

Microbially driven redox cycling of iron (Fe) is an important environmental process that can influence the fate of various subsurface constituents, including carbon, sulfur, nitrate, oxygen, as well as organic and metal/radionuclide contaminants (Tebo and He, 1999; Roden et al., 2004). In circumneutral pH environments, Fe(II)-oxidizing and Fe(III)-reducing bacteria (FeOB and FeRB, respectively) typically thrive in a coupled manner along redox boundaries, where Fe(II) produced via FeRB activity provides an energy substrate for FeOB, and Fe(III) produced via FeOB activity regenerates Fe(III) phases utilized by FeRB (Straub et al., 2001; Roden, 2012). Although aqueous Fe(II) is usually viewed as the main form of Fe(II) utilized by FeOB within redox interfacial environments, recent studies indicate that insoluble primary Fe(II)-bearing mineral phases such as almandine (Chaudhuri et al., 2001) and biotite (Shelobolina et al., 2012b), and secondary Fe-phyllosilicates such as smectite (Shelobolina et al., 2003, 2012a; Benzine et al., 2013) can serve as substrates for FeOB. In addition, the oxidized, Fe(III)-bearing forms of these phases may serve as electron acceptors for FeRB (Kostka et al., 1996, 2002; Dong et al., 2003; Brookshaw et al., 2014), setting up the potential for “solid-state” microbial Fe redox cycling in the vicinity of redox boundaries (Roden, 2012).

A recent study conducted at the Hanford 300 Area site in eastern Washington state recovered several novel subsurface FeOB which are capable of utilizing solid-phase Fe(II) contained in biotite and smectite as energy sources for chemolithotrophic growth (Benzine et al., 2013). The Hanford 300 Area (referred to hereafter as the “300 Area”) subsurface is contaminated with radionuclides (mainly uranium) and nitrate from Cold War-era Pu production (Peterson and Connelly, 2001; Christensen et al., 2004), and as a result there is strong interest in the potential impacts of Fe redox-associated processes on contaminant mobility (Peretyazhko et al., 2012). The 300 Area contains a redox discontinuity at ca. 15–18 m depth, just below the boundary between the coarse-grained Pleistocene Hanford formation (primarily Ice Age cataclysmic flood deposits) and the fine-grained Miocene/Pliocene Ringold formation (primarily of ancestral Columbia River deposits; Lindsey and Gaylord, 1990; Zachara et al., 2007). This redox transition zone (RTZ) has been the subject of a variety of recent work focused on geochemical and microbial interactions and their implications for contaminant mobility and transport (Lee et al., 2012; Lin et al., 2012a,b; Peretyazhko et al., 2012), and was the source of organisms for the silicate mineral-utilizing FeOB study of Benzine et al. (2013). A parallel study from our group (Percak-Dennett and Roden, 2014) examined the hypothesis that microorganisms within and below RTZ could participate in the oxidation of native Fe(II)-bearing phases (primarily dioctahedral smectite with traces of chlorite, Peretyazhko et al., 2012). Contrary to expectations, Fe(II) oxidation was only observed in aerobic, sterile sediments; all other inoculated and sterile reactors amended with oxygen or nitrate showed no Fe(II) loss. Non-sterile sediments consumed significant amounts of nitrate, and were dominated by heterotrophic microbes consuming residual sediment organic carbon. These results indicated that heterotrophic metabolism consumed available oxidants, thereby preventing microbial Fe(II) oxidation. This inference was consistent with previous observations that Ringold Formation sediments contain active heterotrophic microbial communities (Lin et al., 2012a).

Although the above results argued against Fe(II)-driven chemolithotrophy as a major control on redox balance within the 300 Area RTZ, there is nevertheless ongoing interest in the potential for the RTZ to support novel FeOB. This study sought to gain insight into potential 300 Area RTZ FeOB populations by incubating a mixture of quartz sand with Fe(II)-bearing silicate mineral biotite (in comparison to quartz sand alone) *in situ* across (above, within, and below) the RTZ for several months. Our hypothesis was that the sand+biotite mixture would be preferentially colonized by lithotrophic FeOB capable of utilizing Fe(II) in biotite as an energy source. Both the sand+biotite and sand-only *in situ* incubations were free of particulate organic carbon, such that lithotrophic organisms could potentially compete successfully with the heterotrophic communities that otherwise dominate RTZ microbial energy metabolism. *In situ* incubation of the minerals across the RTZ tested the secondary hypothesis that differential colonization of the minerals would not occur below the RTZ where oxidants (oxygen and nitrate) are not available to support FeOB growth. More broadly, the deployment strategy allowed us to examine basic patterns of microbial community composition on the colonized materials in relation to redox and chemical gradients across the RTZ. In addition, the experiment provided an opportunity to evaluate the relative importance of heterotrophic vs. lithotrophic pathways *in situ* within this unique geochemical environment.

## Materials and Methods

### Multi-Level Sampler (MLS) Deployments

In August 2012, a series of 38-mL polypropylene cartridges were loaded with 55 g of either commercial (Accusand, AGSCO Corp., Wheeling, IL, USA) silica sand (ca. 1 mm diameter), or a mixture (1:10 mass ratio) of sand plus fine-grain (ca. 20 μm diameter flakes) biotite, or silica sand only. The biotite was obtained from Ward Scientific and prepared as previously described (Shelobolina et al., 2012b). Biotite was chosen as a Fe(II)-bearing mineral substrate because it is not susceptible to abiotic oxidation by oxygen (or nitrate) on a time scale of weeks to months, and because it was used successfully in a previous study to enrich for FeOB in 300 Area groundwater (Benzine et al., 2013). The cartridges were capped with permeable nylon net filters (20 μm), and deployed in multi-level sampling (MLS) sampling arrays suspended in two different wells at the Hanford 300 Area Integrated Field Research Site (IFRC) as described previously (Lee et al., 2014). Fourteen cartridges were deployed in borehole well 3–24, and 14 cartridges were deployed in well 3–27 [detailed descriptions of these wells can be found in Bjornstad et al. (2009)]. Cartridges were deployed at 10 or 20 cm intervals over depths ranging from 15.3–17.1 m in well 3–24 and 15.5–17.2 m in well 3–27, with sand+biotite or sand-only cartridges alternating with increasing depth. In January 2013, the cartridges were removed, and the contents were transferred to Whirl-Pack bags on ice. The samples were frozen at -80°C upon return to the lab and remained frozen until DNA extraction.

Groundwater samples were collected from MLS arrays deployed prior to the *in situ* mineral deployments, and concentrations of nitrate, total Fe, and total Mn (μM), as well as CH_4_, and H_2_ (ppmv) were determined as previously described (Lin et al., 2012a).

### DNA Extraction and Analysis

#### DNA Extraction from MLS Cartridges

The Whirl-Pack^®^ bags containing the samples were removed from the -80°C freezer and thawed at room temperature. After thawing, the entire content of the bags was transferred to sterile 50 ml Falcon tubes, and the samples were centrifuged at 5000 rpm for 10 min. The supernatant was discarded and the solid-phase material was mixed vigorously to homogenize the samples. Approximately 0.7 g of material was removed for DNA extraction, and the remaining samples were returned to the -80°C freezer.

DNA was extracted from samples using a Mo-Bio PowerSoil^®^ DNA extraction kit (Mo-Bio, Carlsbad, CA, USA) following the manufacturer’s protocol with some modifications. The bead beating tubes (containing the proprietary bead-beating solution) were heated to 70°C prior to the addition of any samples. After heating and sample addition, 80 μl solution C1 was added, and the tubes were heated at 70°C for 10 min with vigorous shaking (14,000 rpm) to aide in cell lysis. As recommended by Mo-Bio, the precipitation steps using solutions C2 and C3 were combined into a single step, using only 100 μL of each, and mixing thoroughly after adding each solution. This procedure is recommended for low-biomass samples to reduce the volume of liquid and the potential for DNA loss. DNA was eluted in a total of 25 μL of Solution C5 and quantified using a Qubit^®^ fluoromoeter (version 1.0, Life Technologies, Inc., Madison, WI, USA). The DNA extracts were stored at -20°C.

#### DNA Extraction from Groundwater Samples

Bulk groundwater samples were collected as previously described (Lin et al., 2012c) before and after the *in situ* mineral deployments for DNA extraction. The pre-MLS deployment groundwater samples were passed through a 47 mm diameter 0.2-μm polyethersulfone filter, and genomic DNA was extracted from half of each membrane filter using a Mo-Bio PowerSoil-HTP 96-well DNA isolation kit according to the manufacturer’s protocol (MoBio Laboratories, Carlsbad, CA, USA). Post-MLS deployment samples were filtered through 0.2 μm Sterivex in-line filters, and DNA was extracted from segments of filters using a Mo-Bio PowerSoil^®^ DNA extraction kit with the modifications described above.

#### Primers and 16S rRNA Gene Amplification

16s rRNA amplicons from each sample were generated using the previously described universal primer set 515f/806r (Bates et al., 2011). Primers containing the template-specific forward or reverse primer, the Roche A (515f primers: 5′-CCATCTCATCCCTGCGTGTCTCCGAC-3′) or B (806r primer only: 5′-CCTATCCCCTGTGTGCCTTGGCAGTC-3′) sequencing adapters, and linker sequence (TCAG) were added to all primers. Appropriate Roche 10-bp MID tags were only added to the 515f primer sets. The final fusion primers were synthesized by Integrated DNA Technologies, Inc. (Coralville, IA, USA).

Samples were amplified with Invitrogen^TM^ Platinum^®^ Taq Supermix (Life Technologies, Grand Island, NY, USA), which contains dNTPs. Each reaction was set up with 5 μL template DNA, 0.1 μL forward and reverse primer (200 nm final concentration), and 45 μL Platinum^®^ Taq Supermix. The following reaction protocol was used to amplify the samples: 94°C for 2 min (initial denaturation) followed by 30 cycles of 94°C for 15 s (denaturing), 55°C for 30 s (annealing), and 72°C for 15 s (extension). A final extension step was conducted at 72°C for 6 min, and the samples were held overnight at 4°C. Each reaction was run on a 1% agarose gel to verify the presence of the amplicon, and to check for spurious amplification products.

#### Sample Pooling and Titanium 454 FLX+ Amplicon Sequencing

Amplicons were pooled in an equimolar ratio, and cleaned using Agencourt^®^ AMPure^®^ XP magnetic beads (Beckman Coulter, Inc., Indianapolis, IN, USA) following the manufacturer’s instructions. The cleanup procedure was repeated six times, as recommended by the University of Wisconsin-Madison Biotechnology Center (UWMBC, James Speers, personal communication). The clean pool was submitted to the UWMBC for quality analysis and Titanium 454 FLX+ sequencing.

#### Sequence Analysis

Raw sequence data was processed using the Quantitative Insights into Microbial Ecology (QIIME) pipeline (http://www.qiime.org, version 1.7.0; Bates et al., 2011). A.sff.txt file was not supplied by the UWMBC, so the mothur script was used to generate it from the raw.sff file (Schloss et al., 2009). This file was used with the QIIME default denoising software (Caporaso et al., 2010). Data processing was conducted using the default QIIME settings: sequences less than 200 bp, with quality scores less than 25, and more than six ambiguous bases were discarded. After quality filtering, the samples were denoised, and operational taxonomic units (OTUs) were picked *de novo* with either 94, 97, or 99% similarity (nominally family-, genus-, and species-level phylogenetic resolution Gillis et al., 2001) using uclust (Edgar, 2010). Putative chimeras were identified and removed with ChimeraSlayer (Edgar et al., 2011), and taxonomy was assigned based on the Greengenes database (Brodie et al., 2006) using the Ribosomal Database Pipeline (RDP) classifier (Wang et al., 2007). The full set of sequences and taxonomic assignments (based on 97% sequence similarity) are provided in Data Sheet 1 of the on-line supplementary material. In addition, the sequences and their taxonomic assignments are available in GenBank submission SUB1010609, accession numbers KT429935 – KT437631 (note that 505 out of the original 8202 OTU sequences were identified as chimeric by NCBI and removed from the list of sequences added to Genbank; the entire set of OTU sequences is included in the supplementary file).

Sequences were aligned using PyNAST (Bates et al., 2011) and a phylogenetic tree for alpha and beta diversity analysis was constructed with FastTree 2 (Price et al., 2010). OTU tables were rarefied at a range of 100–2000 sequences/sample, and ten iterations (without replacement) were conducted. Each rarefaction increased by 100 sequences/sample. OTUs with less than 100 sequences were removed before conducting rarefaction the to smooth the plots. Whole-tree phylogenetic diversity (PD), Chao1, and Observed Species alpha diversity metrics were calculated for each sample, and rarefaction curves were generated in QIIME. The PD-based rarefaction curves were fit by non-linear least-squares regression (Prism GraphPad software, La Jolla, CA, USA) to an equation describing a rectangular hyperbola [*y = ax/(b + x)*], and the estimated maximum number of OTUs (*a* in the fitting equation) was compared to the total number of identified OTUs in order to calculate the degree of saturation for each sample.

Within QIIME, a jackknifed beta diversity analysis was performed after rarefying each sample to 2,000 sequences. Unifrac (Lozupone and Knight, 2005) was used to construct weighted and unweighted Unifrac matrices, and principal coordinates (PCoAs) plots from the results. When further examining abundant taxa, only sequences comprising the 10 most abundant OTUs were considered. To obtain more detailed information about these abundant OTUs, their representative sequences were submitted to BLAST, using the blast-n algorithm (Altschul et al., 1997) with environmental sequences excluded from the database. These results were compared to those obtained when classifying with the RDP pipeline via the Greengenes database.

#### Statistical Analyses

To determine if statistically significant differences or similarities existed among the bacterial communities observed at different depths, or if there were significant differences between deployment types, a Bray–Curtis similarity matrix was constructed from the OTU table using PRIMER for Windows (v.6; Clarke et al., 2006). One-way analysis of similarity (ANOSIM) tests were conducted with 9999 permutations on the untransformed similarity matrix (Clarke, 1993).

CANOCO for Windows (version 4.51) was used to conduct a canonical correspondence analysis (CCA) to determine if geochemical gradients of Fe, NO_3_^-^, and Mn, CH_4_, or H_2_ were responsible for driving the microbial communities observed at different depths as previously described (Beversdorf et al., 2013).

To check for statistical difference in alpha diversity scores (PD, Chao1 matrices), the QIIME script compare_alpha_ diversity.py was used to conduct a Student’s *t*-test and to calculate the *P*-values between groups of mean alpha diversity scores.

## Results

### Groundwater Geochemistry

**FIGURE 1:**
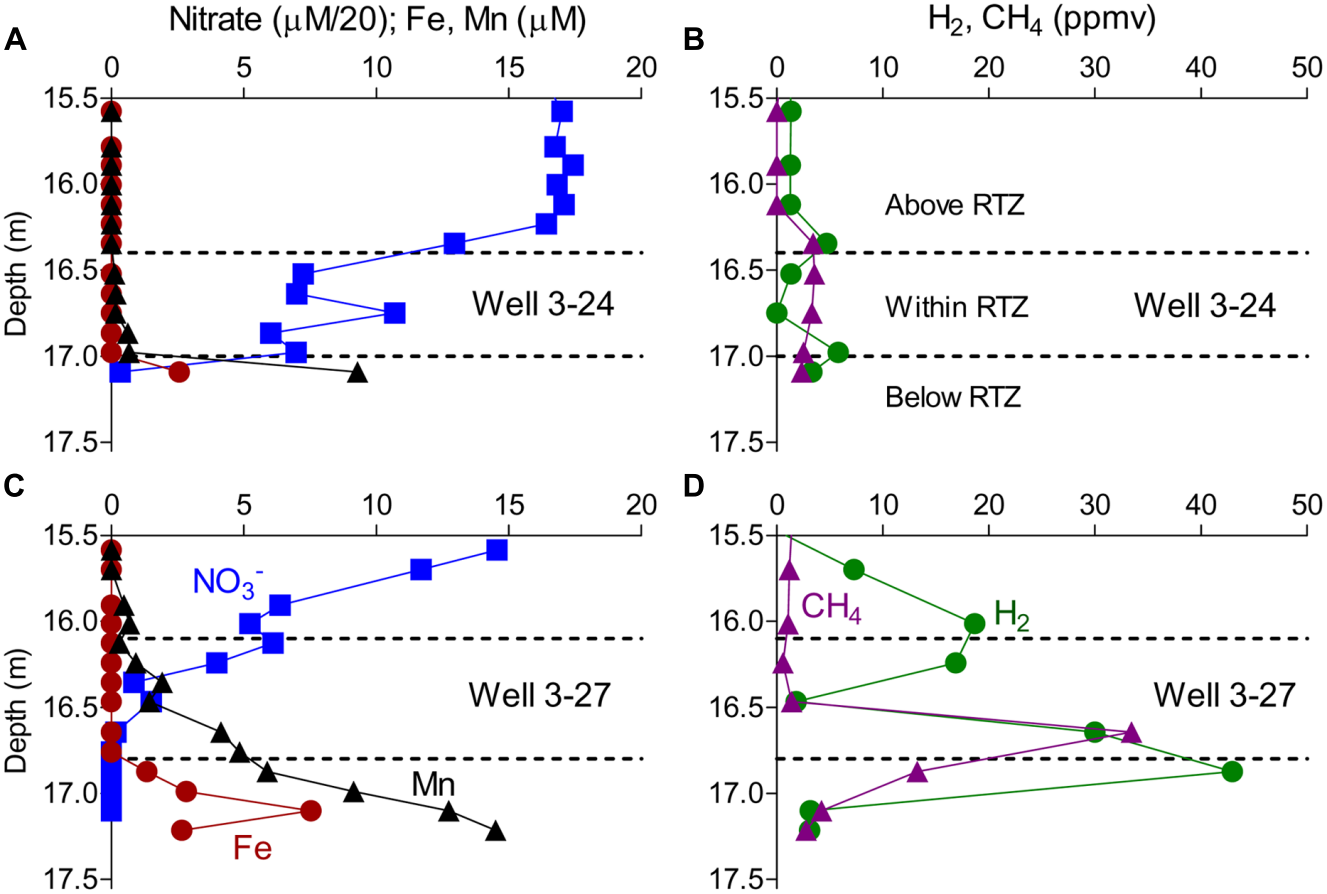
**Concentrations of nitrate, Fe, Mn, CH_4_, and H_2_ measured in wells 3–24 **(A,B)** and 3–27 **(C,D)** measured in August 2012 prior to the *in situ* mineral deployment.** The dashed lines demarcate the depth used to group samples as above, within, or below the RTZ.

Groundwater samples collected in MLS arrays prior to the *in situ* mineral deployments revealed redox gradients comparable to those observed previously (Lin et al., 2012a), with nitrate decreasing to near zero values at the base of the RTZ, and dissolved Fe^2+^ and Mn^2+^ accumulating below that depth (**Figures 1A,C**). Although dissolved oxygen concentrations were not measured in this study, previous studies have shown that oxygen approaches zero ca. 1 m above the depth where nitrate is exhausted (Lin et al., 2012a). The redox gradient was generally steeper in well 3–27, where elevated levels of dissolved methane and hydrogen were also observed below the RTZ (**Figure 1D**).

### 16S rRNA Gene Amplicon Libraries

Thirty-two 16S rRNA gene amplicon libraries were constructed from DNA extracted from 14 MLS cartridges (seven with sand+biotite, seven with sand only) from each of the two wells, plus groundwater samples collected from the two wells before and after *in situ* mineral deployment. After quality filtering and removal of chimeras, a total of 151,477 sequences remained out of a total of 529,551 raw reads, with an average read length of 262 bp. The number of reads per sample ranged from 2235 to 7792, with a mean of 4734. Together the sequences clustered into 8202 OTUs at 97% similarity. The amplicon libraries were grouped by deployment type to explore differences between colonization of sand+biotite or sand alone. In addition, to explore the microbial communities as a function of depth, the samples within each well were grouped into three depth intervals: above the redox transition (15.4–16.4 m in well 3–24, 15.5–16.1 m in well 3–27), within the redox transition (16.5–17.1 m in well 3–24, 16.2–16.8 m in well 3–27) and below the redox transition [17.2 m 3–24 (note: only one sample), 16.8–17.0 m 3–27 in well 3–27]. The groupings were made based on the geochemical gradients as indicated in **Figure 1**. The results of various analyses performed on the grouped data sets are summarized below.

### Phylogenetic Diversity

Faith’s PD (Faith and Baker, 2006) diversity metrics were calculated in QIIME for each of the sample groups after rarefaction (2000 OTUs per sample; and **Table 1**). The post-deployment groundwater samples were the most phylogenetically diverse, and the pre-mineral deployment sample from well 3–24 was also more diverse than any of the samples from the *in situ* mineral deployments. The pre-mineral deployment groundwater sample taken from the 3–27 well was the least phylogenetically diverse of all the samples. There was no difference in PD among the deployment types (sand+biotite or sand only; data not shown). However, there was a decrease in diversity between the mineral deployments above the RTZ and those taken within the RTZ in both wells, and samples from below the RTZ were the least phylogenetically diverse.

**Table 1:** Average Faith’s (Faith and Baker, 2006) phylogenetic diversity (PD) calculated for groundwater and grouped mineral deployment 16S rRNA gene amplicon libraries.

Sample/group	Average phylogenetic diversity (PD)
Post-deployment GW 3–24	84.1
Post-deployment GW 3–27	76.2
Pre-deployment GW 3–24	72.4
Above RTZ 3–27	60.3 ± 3.40
Above RTZ 3–24	54.7 ± 4.10
Within RTZ 3–27	40.5 ± 2.94
Within RTZ 3–24	37.2 ± 4.30
Below RTZ 3–24	32.6
Below RTZ 3–27	29.6 ± 1.84
Pre-deployment GW 3–27	23.4

**FIGURE 2:**
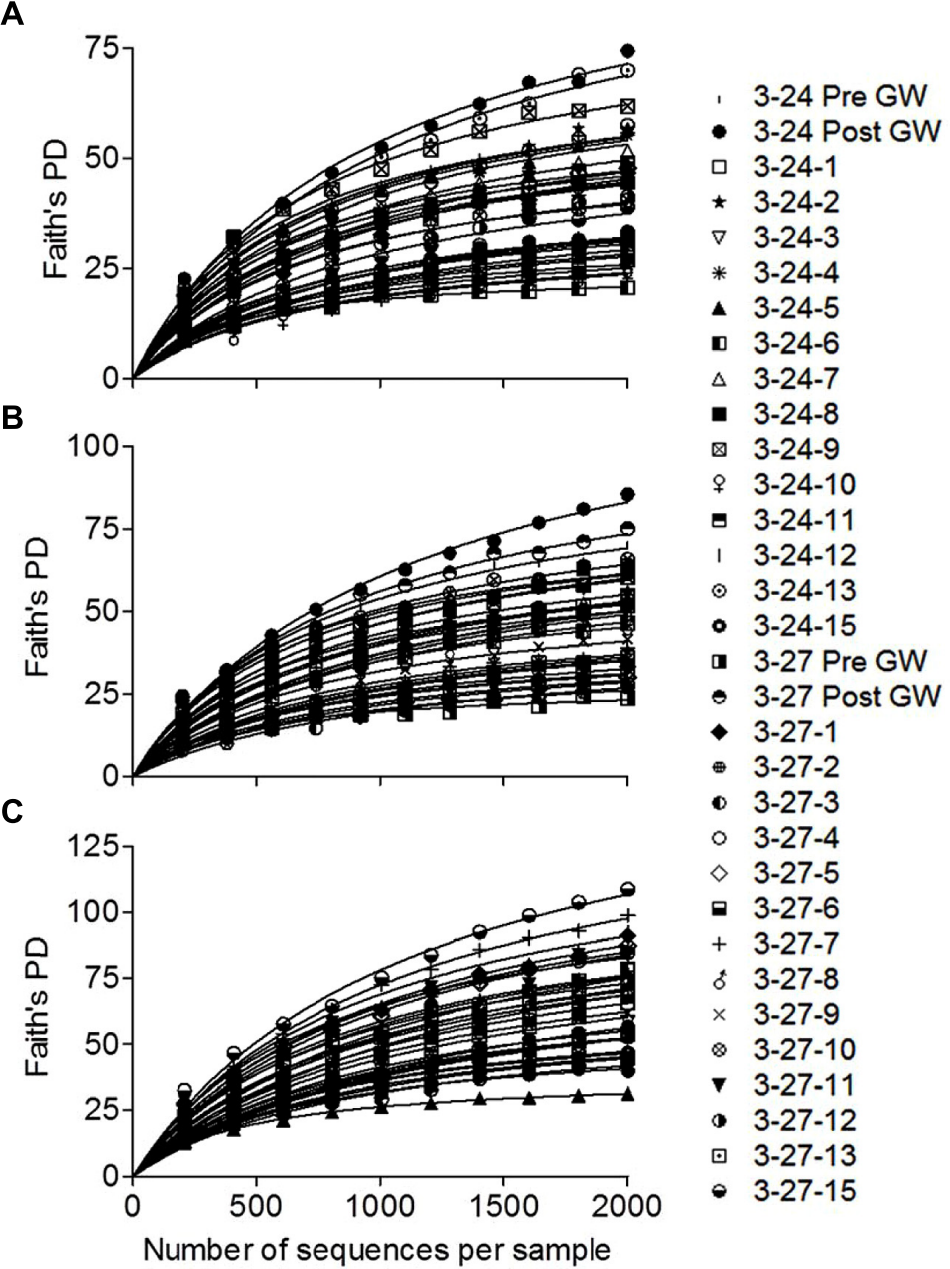
**Rarefaction curves for each of the 16S rRNA gene amplicon libraries generated using PD-based OTU assignments made with either 94 **(A)**, 97 **(B)**, or 99% **(C)** sequence similarity.** Lines represent non-linear least-squares regression fits of the data to an equation describing a rectangular hyperbola *y = ax/(b + x)*, where *a* is the estimated maximum number of OTUs.

*The values presented in the table correspond PD values calculated for the maximum number (*n* = 2000) of sequences included in the rarefaction analysis shown in **Figure 2**. Table is sorted in order of decreasing PD value. Error terms account for the variation among samples within a group; no error was calculated for groups containing only one sample.*

Rarefaction curves were generated for each of the libraries using PD-based OTU assignments made with either 94, 97, or 99% sequence similarity (**Figure 2**). The estimated degree of saturation for the 28 mineral-colonized samples was 75.4 ± 2.9, 71.9 ± 4.1, and 69.0 ± 3.0 for the three levels of similarity. There were no systematic differences in the degree of saturation between the biotite+sand vs. biotite-only samples, or with deployment depth (data not shown). These relatively high levels of saturation permit robust comparisons of mineral-colonized community composition across deployment type and depth. For simplicity, these comparisons were made using the 97% similarity OTU assignments.

### Community Composition across Deployment Type and Depth

**FIGURE 3:**
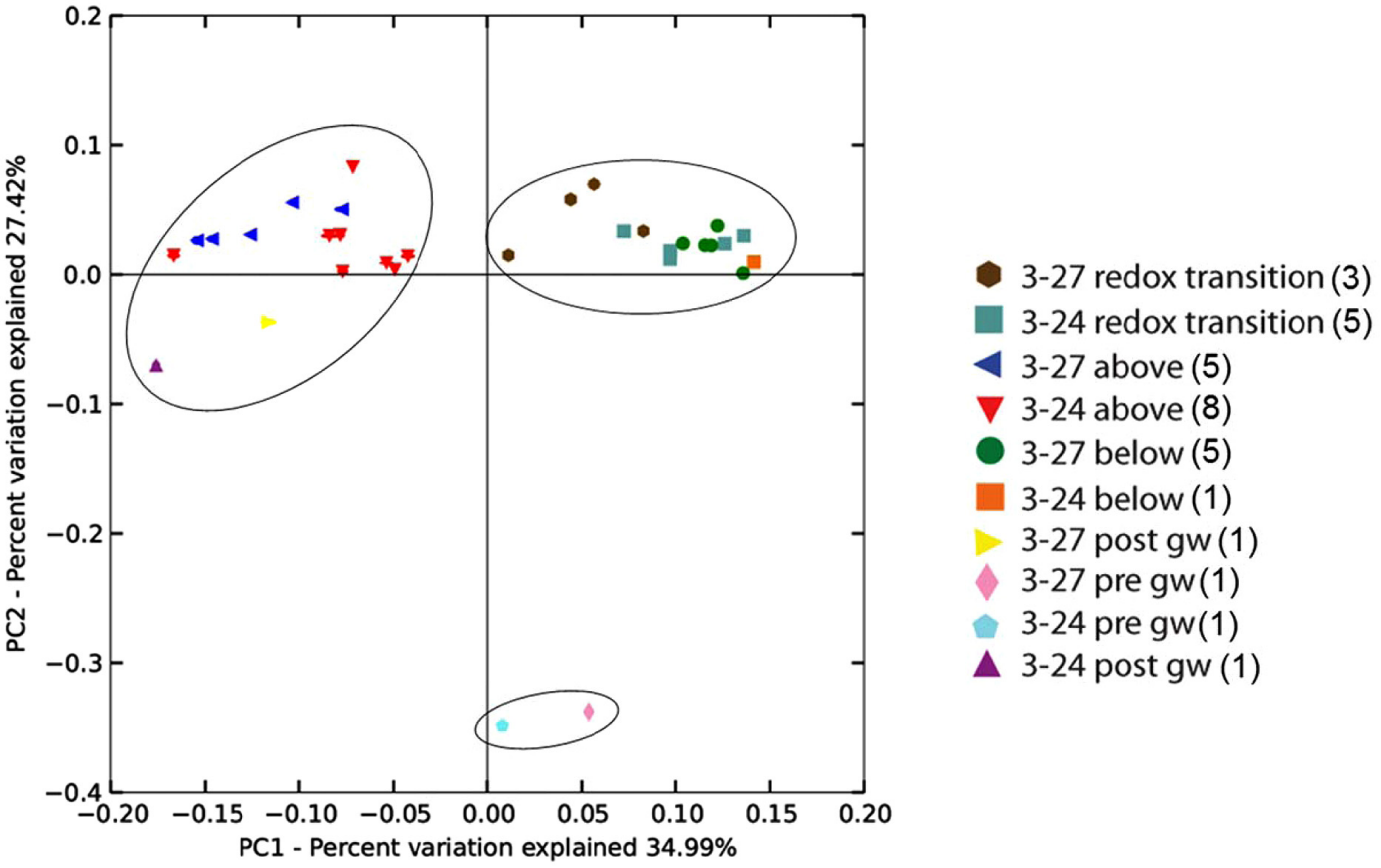
**Weighted Unifrac principal components analysis of 16S rRNA gene sequences from groundwater and grouped mineral deployments constructed from jackknifed beta diversity analysis.** The number of sequences per sample was normalized to *n* = 2000. Values in parentheses in the symbol legend indicate the number of samples included in that groupling. The circles indicate the three clusters of samples, which were determined to be statistically different with ANOSIM (*R* = 0.818). There was no significant similarity between samples from different deployment types (*R* = 0.314).

Weighted and unweighted Unifrac (Caporaso et al., 2010) matrices were constructed to determine if the microbial communities were similar across deployment type (sand+biotite vs. sand only) or depth (above, below, or within the RTZ). The OTU table was rarefied to a depth of 2000 sequences prior to calculating Unifrac distances, and PCoA plots were constructed from the weighted Unifrac matrices. The PCoA plot of the weighted Unifrac matrix (**Figure 3**) indicated that there was no difference between the microbial communities associated with mineral deployment type, indicating that differential colonization of cartridges containing biotite did not occur or was below the resolution limits of our data set.

Principal coordinate analysis revealed two distinct sample clusters based on depth, together with one loose cluster comprising the pre-mineral deployment groundwater sample. Samples from above the RTZ in both wells formed a cluster with the post-deployment groundwater samples, and samples from within and below the RTZ formed a distinct cluster, although samples from within the RTZ in well 3–27 (brown hexagons in **Figure 3**) did not cluster as tightly as the other samples, suggesting some similarity with the samples from above the RTZ. The slight difference in RTZ samples from each well is likely due to the differences in sampling depths within each well, as some of the RTZ samples in well 3–24 were from the same depth as the below RTZ samples in well 3–27. There was no significant difference between the communities between each well.

### Abundant Taxa Present in all Depth Intervals

**FIGURE 4:**
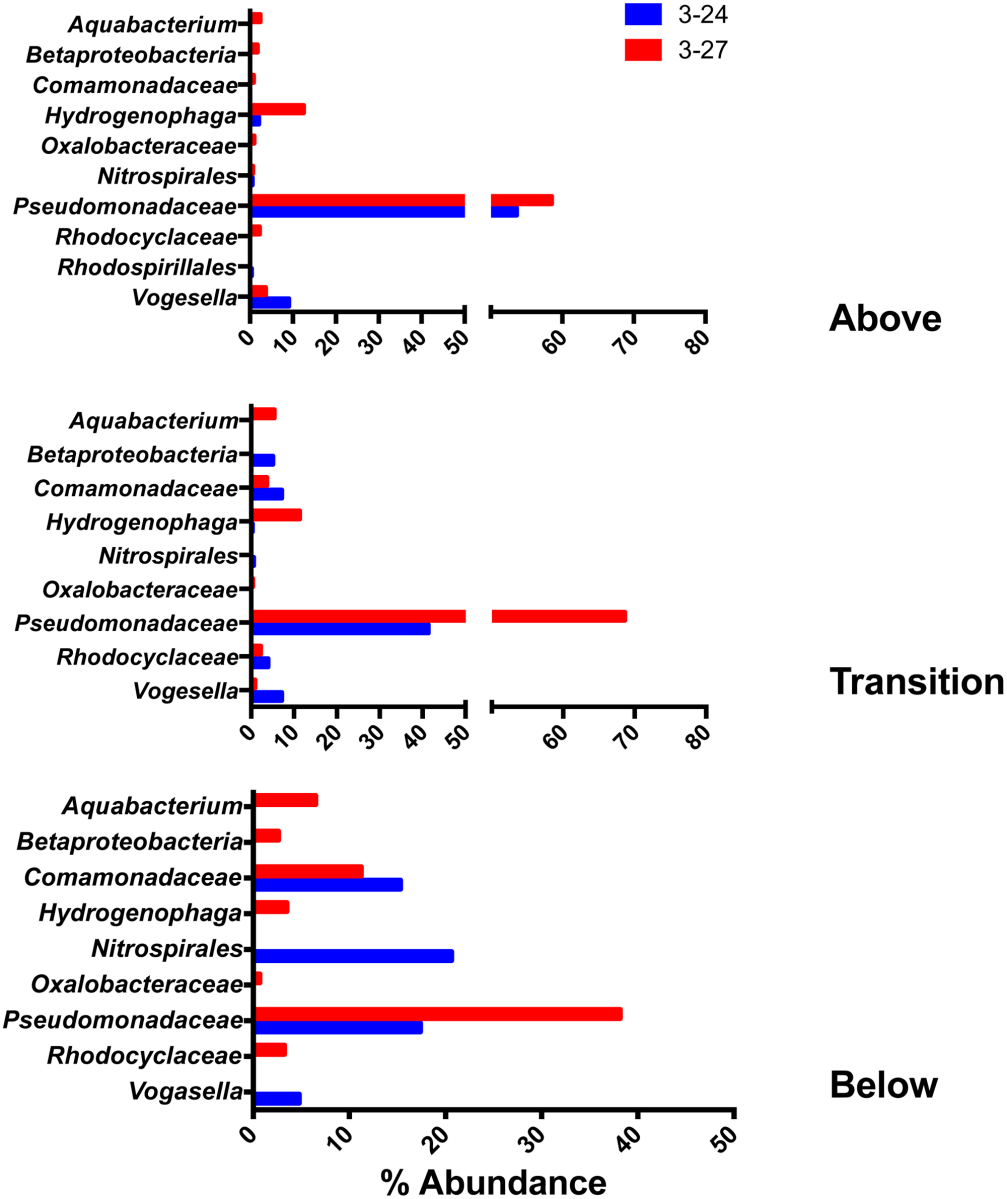
**Top 10 most abundant taxa detected in all three of the depth interval groupings (above, within, and below the RTZ)**.

The 10 most abundant (percentage-wise) OTUs from each sample were pooled within their respective depth intervals (above, within, or below the RTZ), and the wells were evaluated independently due to the slight differences in deployment depth in each well. The 10 most abundant OTUs accounted for an average of 88.0 ± 6.1% and 89.7 ± 4.5% of total OTUs (averaged across all depths) for wells 3–24 and 3–27, respectively. In some cases, RDP classified independent OTUs at the same level, and these OTUs were grouped together. **Figure 4** highlights the taxa that were found in all depth intervals.

Members of the *Pseudomonadaceae* were the most abundant taxa at all depth intervals in both wells. They were also the only taxa detected in both wells at all depth intervals. Two different OTUs (see **Table 2**) comprised anywhere from 17% (well 3–24, below the RTZ) to 69% (well 3–27, within the RTZ) of the reads within a given depth interval. Other abundant OTUs detected at all depths (in one or the other of the two wells) included *Aquabacterium, Betaproteobacteria, Comamonadaceae, Hydrogenophaga, Nitrospirales, Oxalobacteraceae, Rhodocyclaceae*, and *Vogesella*. The abundance of *Hydrogenophaga* was considerably lower at all depths compared to *Pseudomonadaceae*, with a maximum abundance of 2.8% in well 3–24, and 12.5% in well 3–27. The abundance percentages of this group decreased with depth. *Vogesella* abundance also decreased in abundance with depth, from 9.1% to 4.8% in 3–24, and 3.7% to 0% in 3–27. *Aquabaterium* and *Oxalobacteraceae* were only detected in well 3–27. At the shallowest depths, *Aquabaterium* comprised only 2.4% the reads, but this increased to 6.5% below the RTZ. *Oxalobacteraceae* comprised ca. 1% abundance at all depths.

**Table 2:** Potential chemolithotrophic taxa identified in the 16S rRNA gene amplicon libraries.

Well	QIIME assignment	% of total reads^a^	BLAST match	Accession No.	Similarity (%)	Physiology	Reference
3–24	*Rhodocyclaceae*	2.5 (B)	*Leptothrix ochracea* SAG^b^	HQ290491.1	98	Fe(II) oxidation	Fleming et al. (2011)
3–24	*Thiobacillus*	2.0 (T), 5.2 (B)	*Thiobacillus thioparus*	DQ451827.1	100	S(-II) oxidation	Sattley and Madigan (2006)
3–24	*Nitrosopumilus*	3.2 (A), 10.7 (T)	Candidatus *Nitrosopumilus maritimus*	HQ331116.1	99	NH_4_^+^ oxidation	Jung et al. (2011)
3–24	*Cenarchaeaceae*	4.25 (A), 4.95 (T)	Candidatus *Nitrosopumilus* sp. strain NF5	CP011070.1	92	NH_4_^+^ oxidation	Jung et al. (2011)
3–24	*Alteromonadaceae*	0.3 (A), 1.2 (T)	*Thioprofundum hispidum*	NR_112620.1	94	S(-II) oxidation	Mori et al. (2011)
3–24	*Gallionella*	1.1 (B)	*Gallionella* sp. strain JA52	KC677661.1	96	Fe(II) oxidation	Tischler et al. (2013)
3-24	*Bradyrhizobium*	0.8 (A)	*Bradyrhizobium* sp. strain 22	KF800709.1	100	Fe(II) oxidation	Benzine et al. (2013)
3–24	*Elusimicrobiales*	0.41 (A)	*Halothiobacillus kellyi* strain BII-1	NR_025030.1	85	S(-II) oxidation	Sievert et al. (2000)
3–24	*Elusimicrobiales*	0.3 (A)	*Halothiobacillus* sp. strain ATSB12	EF397575.1	84	S(-II) oxidation	Anandham et al. (2008)
3–24	*Nitrospira*	0.2 (A), 0.9 (T)	*Nitrospira moscoviensis* strain NSP M-1	NR_029287.1	99	NO_2_^-^ oxidation	Ehrich et al. (1995)
3–27	*Hydrogenophaga*	12.5 (A), 11.0 (T), 3.5 (B)	*Hydrogenophaga* sp. strain AH-24	AB300163.1	98	H_2_ oxidation	Yoon et al. (2008)
3–27	*Nitrospira*	0.7 (A)	*Nitrospira moscoviensis* strain NSP M-1	NR_029287.1	99	NO_2_^-^ oxidation	Ehrich et al. (1995)
3–27	*Nitrosopumilus*	14.9 (A), 0.8 (T)	Candidatus *Nitrosopumilus maritimus*	HQ331116.1	99	NH_4_^+^ oxidation	Jung et al. (2011)
3–27	*Cenarchaeaceae*	1.6 (A)	Candidatus *Nitrosopumilus* sp. strain NF5	CP011070.1	92	NH_4_^+^ oxidation	Jung et al. (2011)
3–27	*Elusimicrobiales*	2.5 (A), 0.5 (T)	*Halothiobacillus kellyi* strain BII-1	NR_025030.1	85	S(-II) oxidation	Sievert et al. (2000)
3–27	*Bradyrhizobium*	0.9 (A)	*Bradyrhizobium* sp. strain 22	KF800709.1	100	Fe(II) oxidation	Benzine et al. (2013)
3–27	*Rhodocyclaceae*	1.4 (B)	*Leptothrix ochracea* SAG	HQ290491.1	98	Fe(II) oxidation	Fleming et al. (2011)

*^a^A = above redox transition zone (RTZ); T = within RTZ; B = below RTZ.*

*^b^SAG = Single Amplified Genome.*

*Nitrospirales* were the only other group detected in all depth intervals (aside from the *Pseudomonadaceae)* that were present at >20% abundance in at least one depth interval. They were detected in both wells above the RTZ, and in one sample from below the RTZ in well 3–24 where their relative abundance was ca. 20%. At all other depths, their abundance remained relatively low (≤2%). *Comamonadaceae* were detected both within and below the RTZ in both wells, and in one sample above the RTZ in well 3–27. A group of taxa classified by RDP only at the phylum level as *Betaproteobacteria* were found above and below the RTZ in well 3–27, and was present in both wells within the RTZ. This group of OTUs comprised ca. 0–5% of the abundance in both wells, but was most abundant within the RTZ in well 3–24 RT at 5%. BLAST searches indicated that this group was a 95% match to *Burkholderia* sp. The *Rhodocyclaceae* group was present in the same pattern of depth intervals at the *Betaproteobacteria* group, and, as above, there was no relationship between abundance and depth, with percentages ranging from 0 to 4%.

### Taxa Abundant in some Depth Intervals

**FIGURE 5:**
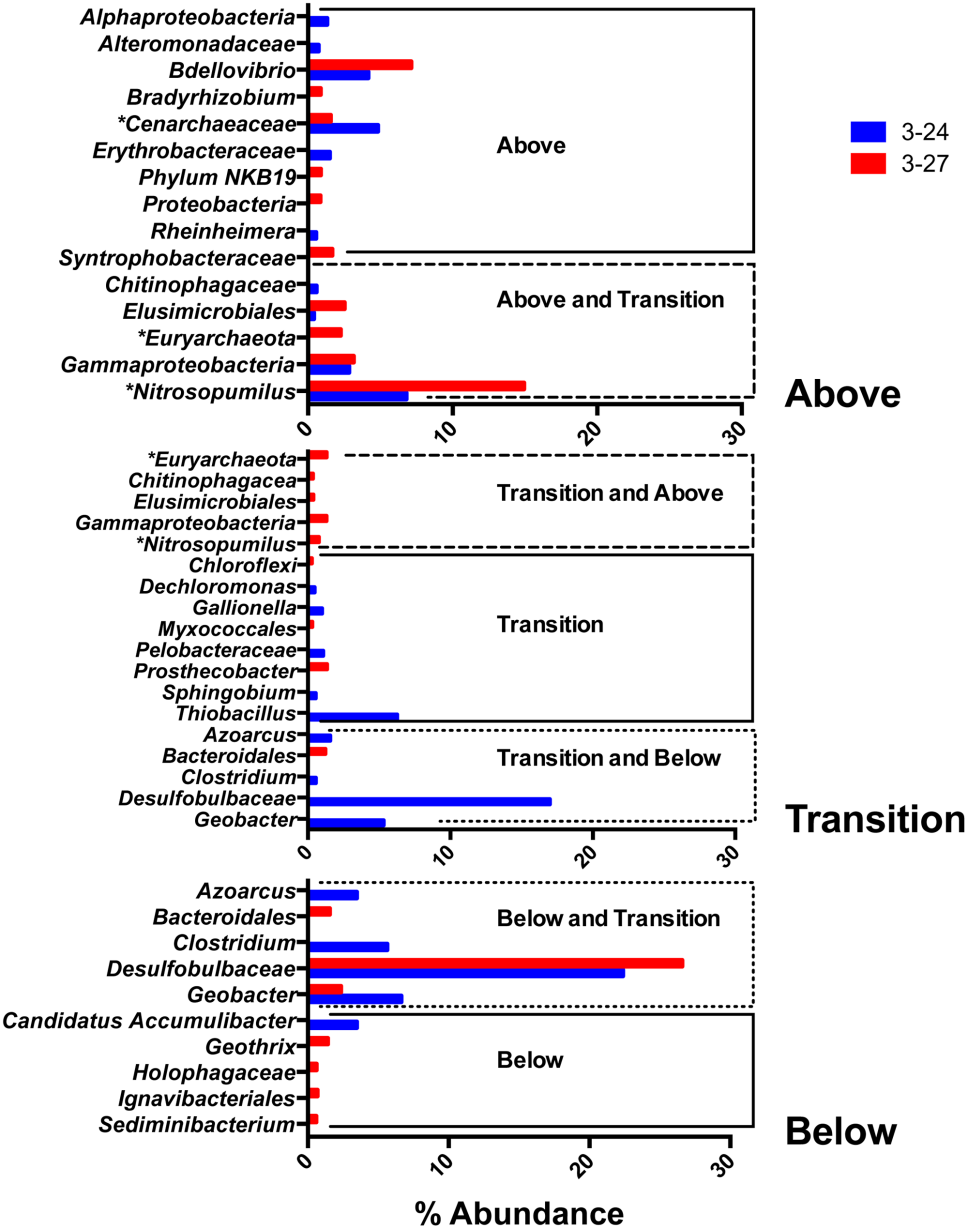
**Top 10 most abundant taxa detected in one or two of the depth interval groupings (above, within, and below the RTZ)**.

The remaining abundant taxa were detected in one or two of the depth interval groupings, but not all three. The abundance of these taxa are presented in **Figure 5**. None of these groups, with the exception of a group classified by RDP as *Desulfobulbaceae*, were detected at a greater than 20% abundance.

#### Taxa above the RTZ

OTUs related to the predatory bacterial genus *Bdellovibrio* were abundant in colonized minerals from above the RTZ. Other taxa detected solely above the RTZ included groups classified as *Bradyrhizobium* and the candidate phylum NKB19. These groups were only detected in well 3–27 at low abundance.

#### Taxa above and within the RTZ

Of the taxa detected both above and within the RTZ, the most abundant were a collection of OTUs classified by RDP as *Gammaproteobacteria*, at 2.85% in well 3–24 and 3.15% in well 3–27. A number of archaeal taxa were detected above and within the RTZ, including a group classified as *Cenarchaeaceae*, which was present above the RTZ in both wells. Two other archaeal taxa belonged to the *Euryarchaeota* family and the *Nitrosopumilus* genus. *Euryarchaeota* were only present in well 3–27, whereas *Nitrosopumilus* was detected in both wells above the RTZ, and in 3–27 within the RTZ. Of the organisms detected only above the RTZ, *Nitrosopumilus* was detected at the greatest abundance at 15% in well 3–27. No archaeal taxa were detected below the RTZ.

#### Taxa within the RTZ

Among the taxa detected exclusively within the RTZ, only one group, classified as *Thiobacillus*, was detected at an abundance >5% (well 3–24 only). In well 3–27, the most abundant organism was a group classified as *Prosthecobacter*, at 1.35%.

#### Taxa within and below the RTZ

Of the taxa detected within and below the RTZ, all but one group were detected exclusively in well 3–24, including the most abundant taxa detected within the RTZ, a group classified as *Desulfobulbaceae* (16.7%). Other abundant groups detected in both these depth intervals include *Clostridium* and *Geobacter*, at 0.56 and 5.32%, respectively. The only taxa detected both within and below the RTZ in well 3–27 was a group classified as *Bacteroidales* (1.23%).

#### Taxa below the RTZ

The *Desulfobulbaceae* group described in the previous paragraph was the most abundant group detected below the RTZ at 27 and 22.4% in wells 3–27 and 3–24, respectively. The *Clostridium* and *Geobacter* groups described above were present in well 3–24 at 5.6 and 6.6%, and the *Geobacter* group was also present in well 3–27 at 2.25%. Among the taxa unique to below the RTZ, *Geothrix, Holophagaceae, Ingavibacteriales*, and *Sediminibacterium* were detected in well 3–27, and a group classified as *Candidatus Accumulibater* was detected in well 3–24.

### Abundant Taxa in Groundwater Samples

**FIGURE 6:**
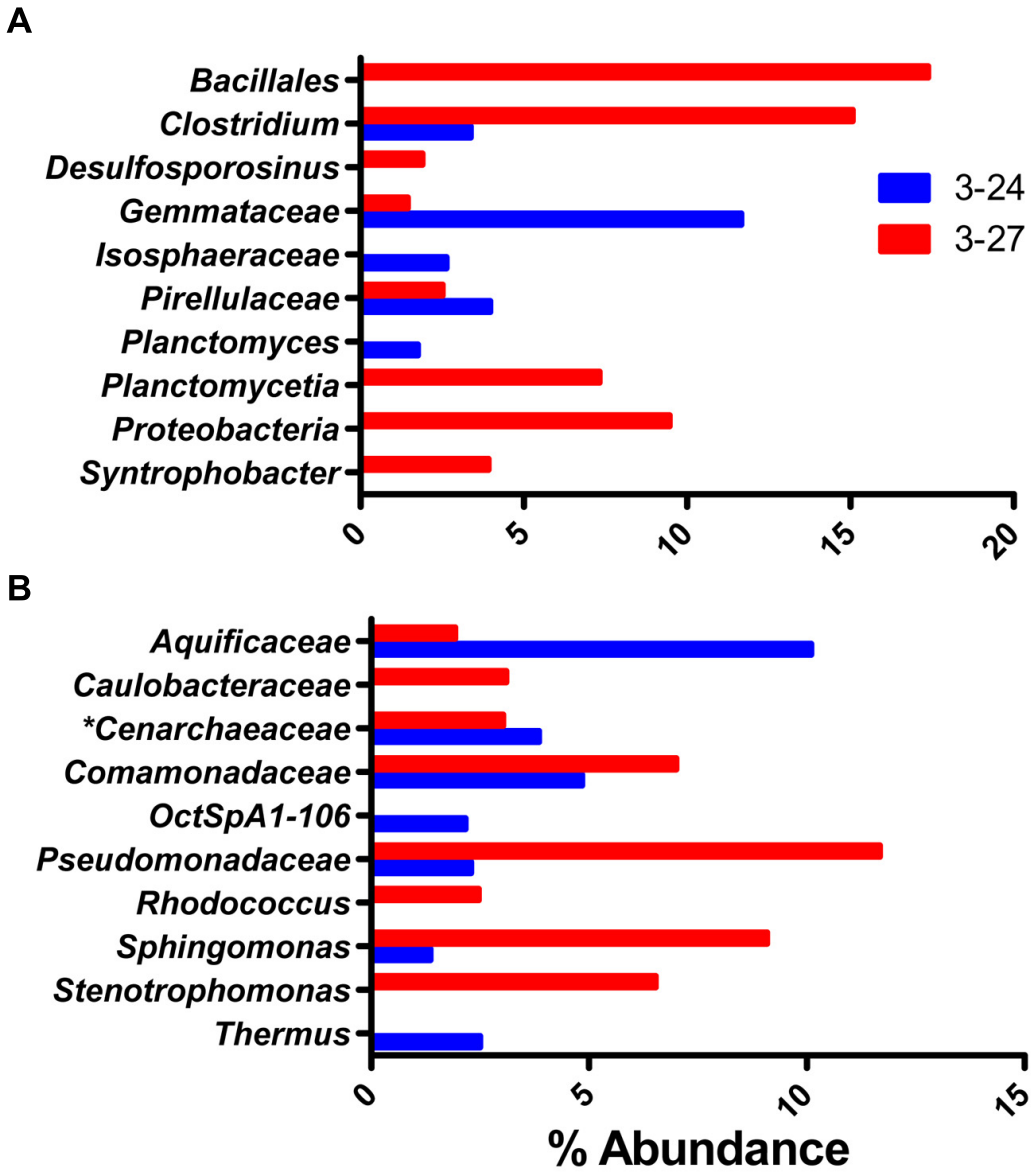
**Top 10 most abundant taxa detected in one or two of the groundwater wells prior to **(A)** or after **(B)** the *in situ* mineral incubation experiment**.

The most abundant OTUs present in the groundwater samples were considerably different than the abundant taxa observed in the colonized minerals. In addition, there were no overly dominant taxa, with the most abundant OTUs in each sample representing less than 20% of the reads (**Figure 6**), and the top 10 most abundant OTUs accounting for only 39.5 ± 15.9% of total OTUs as compared to ≥88% for the mineral deployments. Although the post-mineral deployment groundwater communities clustered loosely with those that colonized minerals above RTZ, the pre-mineral deployment communities were distinct from all other samples. The pre-mineral deployment sample from well 3–24 was the most phylogenetically diverse sample recovered (**Table 1**), and contained organisms (e.g., *Bacillales, Desulfosporosinus, Gemmataceae, Isosphaeraceae, Pirellulaceae*, and *Planctomycetia*) that were rare or not present in the other libraries.

## Discussion

The original goal of this study was to evaluate the hypothesis that organisms related to known FeOB would preferentially colonize mixtures of sand and the Fe(II)-bearing mineral biotite compared to sand alone when the minerals were suspended in Hanford Area 300 groundwater. This hypothesis arose from previous *in situ* biotite incubation studies which resulted in isolation of several different FeOB from the RTZ at the 300 Area site (Benzine et al., 2013). A secondary hypothesis was that differential colonization of the sand+biotite vs. sand-only deployments would disappear going across the RTZ, since oxidants (oxygen and nitrate) capable of supporting FeOB growth are absent below the RTZ. Contrary to these hypotheses, the 16S rRNA gene amplicon sequencing results indicated no difference in microbial community composition between the sand+biotite vs. sand-only treatments regardless of the depth at which the minerals were suspended in the groundwater (**Figure 3**). Instead, the sequence data demonstrated a predominance of heterotrophic taxa at all depth intervals. In addition, distinct depth-dependent changes in certain components of the microbial community were observed, indicating proliferation of anaerobic taxa within and below the RTZ, and the presence of chemolithotrophic taxa in the vicinity of the RTZ. Details related to these findings are presented below. Before doing so, however, it is important to acknowledge the possibility that the lack of difference between the sand+biotite vs. sand-only microbial communities is an artifact of the relatively short deployment interval, i.e., that preferential colonization of the biotite by lithotrophic organisms might have occurred if the minerals had undergone more lengthy *in situ* incubation. Put another way, it seems possible that the observed colonization represented an evolving response to environmental conditions, where the sampled community responded to the presence of readily available heterotrophic energy sources (e.g., DOC; see below) rather than the long-term presence of biotite as a lithotrophic energy source. A related issue (kindly raised by a reviewer of this paper) is that there could have been competition for mineral surface area as a resource for growth and colonization, with the heterotrophs having an energetic advantage (e.g., in terms of growth yield) compared to chemolithotrophs. This argument provides a simple explanation for why Fe(II)-oxidizers were not dominant members of the communities established on the emplaced minerals.

### Heterotrophic Microbial Communities on Colonized Minerals

Members of the *Pseudomonadaceae* were the most abundant taxa detected in the colonized mineral samples (**Figure 4**). BLAST searches revealed that all of the *Pseudomonadaceae* assignments from the QIIME pipeline belong to the genus *Pseudomonas*. *Pseudomonas* sp. are known for their propensity to adhere to surfaces (Moore et al., 2006), and this together with their significant abundance in 300 Area groundwater [**Figure 6B**; see also Lin et al. (2012c)] and capacity to utilize a wide variety of organic substrates (Moore et al., 2006) may account for their predominance on the colonized materials. Other predominant taxa associated with the colonized minerals included known aerobic and/or facultatively anaerobic taxa such as *Aquabacterium, Comamonadaceae, Vogesella* (**Figure 4**); and known anaerobic taxa such as *Clostridium, Desulfobulbaceae, Geobacter, Bacteroidales* within and below the RTZ (**Figure 5**).

The observation that heterotrophic, rather than lithotrophic taxa, were most abundant on the incubated minerals is consistent with previous results indicating the capacity for heterotrophic metabolism within Hanford 300 Area sediments and groundwater (Lee et al., 2012; Lin et al., 2012a; Konopka et al., 2013; Percak-Dennett and Roden, 2014), as well as the predominance of heterotrophic taxa within 300 Area sediments and groundwater (Lin et al., 2012b,c). Hanford 300 Area groundwater contains low (≤1 ppm) but detectable quantities of dissolved organic carbon (DOC levels measured in bulk samples pumped from wells 3–24 and 3–27 prior to the *in situ* mineral deployment were approximately 0.6 ppm) which likely supported heterotrophic colonization of the mineral substrates. A recent single-cell genomic study of *Pedobacter* sp. present in 300 Area groundwater demonstrated the presence of a wide range of both intra and extracellular carbohydrate-active enzymes that may enable aerobic degradation of polymeric substrates as well as utilization of more labile sugars such as mannose and fucose (Wilkins et al., 2014). Although only a few OTUs related to *Pedobacter* were present in our libraries, it seems reasonable to assume that the various heterotrophic taxa that colonized the minerals possessed analogous metabolic capabilities.

The relatively high abundance of the aerobic predatory bacterium *Bdellovibrio* in samples from above the RTZ is consistent with the presence of a robust aerobic heterotrophic microbial community. Reports of *Bdellovibrio* in aquifer environments are rare, although Hutchens et al. (2004) found evidence for *Bdellovibrio* predation on methanotrophic bacteria in a groundwater-fed cave. The low abundance of *Bdellovibrio* in the groundwater samples (data not shown) compared to the colonized minerals suggests that these organisms became enriched in the MLS cartridges via predation on organisms attached to the minerals. More broadly, the large taxonomic differences between the most abundant mineral-associated vs. groundwater microbiota indicates that the presence of the particle attachment sites fundamentally altered microbial community structure. It should be noted, however, that fluid advection could have had an independent impact on groundwater community composition during the *in situ* mineral incubation period, i.e., by driving dispersal of taxa among differing depths or locations within the riparian corridor aquifer (Stegen et al., 2013).

### Shifts in Microbial Community Composition across the RTZ

Principal coordinate plots of weighted Unifrac data revealed two distinct clusters of samples based on deployment depth, where communities above the RTZ were distinct from those within and below the RTZ (**Figure 3**). The observed shift in community composition going across the RTZ is consistent with alpha diversity analyses that indicated PD decreased with depth (**Figure 2** and **Table 1**). A previous study noted differences in microbial communities at depths near the RTZ in 300 Area sediments (Lin et al., 2012b), likely due to the redox gradients present in the upper Ringold formation. Our results clearly demonstrate proliferation of the Fe(III)- and/or sulfate-reducing taxa *Desulfobulbaceae* (Holmes et al., 2004) and *Geobacter* (Lovley et al., 2011) [as well as other less abundant Fe(III)-reducing taxa such as *Geothrix* (Coates et al., 1999), *Ignavibacteriales* (Podosokorskaya et al., 2013), and *Pelobacteraceae* (Lovley et al., 1995)] on the colonized minerals within and below the RTZ. These results are consistent with qPCR studies which showed a peak in *Geobacteraceae* 16S rRNA and *dsrA* genes in the vicinity of the RTZ (Lin et al., 2012a), as well as with the appearance of the minerals upon recovery from the MLS arrays: materials from within and (more notably) below the RTZ had a distinct black color indicating accumulation of iron monosulfides (FeS). It is likely that Fe(III) oxides were produced from oxygen or nitrate-driven Fe(II) oxidation (see below) in the vicinity of the RTZ (see **Figure 1**), and that these were subsequently utilized by Fe(III)-reducers, generating Fe(II) that reacted with dissolved sulfide produced by sulfate-reducers to form the black FeS coatings on the sand grains. As observed previously (Lin et al., 2012a), dissolved sulfide concentrations were at or below detection (a few μM) in MLS groundwater samplers deployed prior to the *in situ* mineral incubation experiment, which is consistent with rapid scavenging of sulfide through reaction with Fe(II). Relatively low but detectable quantities of iron-sulfide minerals are present below the RTZ in Hanford 300 Area sediments (Peretyazhko et al., 2012; Percak-Dennett and Roden, 2014), and sediment incubation studies have demonstrated the capacity for Fe(III) and sulfate reduction driven by oxidation endogenous organic carbon sources in fine-grained 300 Area sediments (Lee et al., 2012; Percak-Dennett and Roden, 2014). Hence, it appears that the microbial communities that arose on the *in situ*-incubated minerals mirrored the composition and metabolic activity of communities in the sediments themselves.

### Potential for Lithotrophic Microbial Metabolism within and above the RTZ

Although heterotrophic organisms clearly dominated the colonized minerals, several putative lithotrophic taxa were detected in significant abundance above, within, and in a few cases below the RTZ (**Table 2**). BLAST searches were conducted on these OTUs to gain further insight into the potential physiological capacities of the specific taxa detected in the libraries. Although caution must exercised in inferring physiology based on 16S rRNA gene similarity (Achenbach and Coates, 2000), this basic approach pioneered by Pace (1996, 1997) remains standard practice in microbial ecology. We focused on pure culture relatives of the clone sequences, for which reasonable physiological inferences could be made.

As expected given the requirement for oxidants such as oxygen and/or nitrate, the most abundant lithotrophic taxa were detected above and within the transition, including *Nitrosopumilus*, an aerobic, chemolithoautotrophic, NH_4_^+^ oxidizing *Crenarchaeota* (*Thaumarchaeota*) known to occur in both marine (Könneke et al., 2005) and soil (Jung et al., 2011) environments; and *Hydrogenophaga*, an aerobic, chemolithoautotrophic, H_2_-oxidizing *Betaproteobacterial* taxon (Willems et al., 1989; Yoon et al., 2008) that is present in a variety of natural and engineered (e.g., waste water treatment) environments (Schwartz and Friedrich, 2006). Although NH_4_^+^ measurements are not available for the Hanford 300 Area groundwaters, the presence of substantial nitrite in the vicinity of the RTZ is consistent with nitrification activity, as is the detection of nitrite-oxidizing relatives of *Nitrospira* (**Table 2**). It must acknowledged, however, that the presence of nitrite could be the result of partial denitrification rather than nitrification. In the case of H_2_ oxidizers, the presence of *Hydrogenophaga* makes sense as the geochemical data suggested an overall upward flux of dissolved H_2_ into the RTZ (**Figure 1**). A recent study recovered an autotrophic H_2_-oxidizing, nitrate-reducing *Acidovorax* sp. from *in situ*-incubated sand (using the same methods employed in this study) in 300 Area groundwater (Lee et al., 2014), which is consistent with the likely role of H_2_ metabolism in energy metabolism in the vicinity of the RTZ. A recent study demonstrated the ability of nitrite-oxidizing *Nitrospira* to grow autotrophically with H_2_ (Koch et al., 2014), and thus it seems possible that some of the *Nitrospira* relatives detected in 16S libraries could also have participated in H_2_ metabolism.

Amplicons related to three known chemolithotrophic FeOB were present at relatively low but easily detectable numbers in the 16S rRNA gene libraries, including sequences related to (1) a single amplified genome (SAG) of *Leptothrix ochracea* from a freshwater Fe seep in Maine, USA (Fleming et al., 2011); (2) *Gallionella* sp. strain JA52 isolated from mine water treatment plant in eastern Germany (Tischler et al., 2013); and (3) *Bradyrhizobium* sp. strain 22, one of the chemolithotrophic FeOB recently isolated from groundwater and sediments from the 300 Area RTZ (Benzine et al., 2013). It seems feasible that these organisms could thrive via oxidation of soluble Fe(II) produced below the RTZ (see **Figures 1A,C**).

Amplicons related to two reduced sulfur-oxidizing taxa were also detected in the 16S rRNA gene libraries, including strains of *Thiobacillus thioparus* isolated from stratified Lake Fryxell in Antarctica (Sattley and Madigan, 2006), and strains of *Halothiobacillus* sp. recovered from a shallow-water hydrothermal vent in the Aegean Sea and from various crop plant rhizosphere soils from Korea (Anandham et al., 2008). Although dissolved sulfide (HS^-^) levels were below detection in our MLS samples, Lin et al. (2012a) documented substantial HS^-^ (up to ca. 40 μM) in groundwaters a few m below the RTZ. Lithotrophic sulfide oxidation could potentially contribute (along with reactions with sediment Fe phases) to scavenging of sulfide entering the RTZ from deeper groundwaters.

Collectively the above findings indicate that chemolithotrophic pathways, fueled by reduced compounds generated via anaerobic heterotrophic metabolism within and below the RTZ, are likely to play a significant role in elemental cycling in the vicinity of the RTZ.

## Conclusion

An *in situ* mineral incubation experiment was conducted in the vicinity of a subsurface RTZ at the Hanford 300 Area site (Richland, WA, USA) to examine the hypothesis that FeOB would preferentially colonize mixtures of sand and the Fe(II)-bearing mineral biotite compared to sand alone. The study was motivated by previous work that documented the presence of Fe(II)-oxidizing bacteria capable of oxidizing structural Fe(II) in primary silicate and secondary phyllosilicate minerals in sediments and groundwater from the 300 Area (Benzine et al., 2013). In contrast to expectations, pyrosequencing of 16S rRNA gene amplicons from the colonized minerals showed no significant difference in community composition between the two treatments. However, the culture-independent analysis revealed interesting aspects of microbial community composition in the vicinity of the RTZ, including an overall predominance of heterotrophic taxa, and a clear shift toward anaerobic Fe(III)- and sulfate-reducing taxa below the RTZ. These results are consistent with previous studies of microbial community composition and activity in the vicinity of the RTZ, and reinforce the view that heterotrophic metabolism is likely to play a key role in maintenance of the redox boundary over long time scales in Area 300 sediments (Percak-Dennett and Roden, 2014). Despite the predominance of heterotrophic taxa, significant numbers of OTUs related to known NH_4_^+^, H_2_, Fe(II), and HS^-^ oxidizing taxa were detected in the 16S rRNA gene libraries. The activity of these organisms, fueled by reduced compounds generated via anaerobic heterotrophic metabolism within and below the RTZ, could play a significant role in elemental cycling in the vicinity of the RTZ. The inferred coupling of heterotrophic and lithotrophic metabolic pathways has important implications for the fate and transport of redox-sensitive metals and radionuclides such as U and Tc across the Area 300 RTZ and other analogous environments.

## Conflict of Interest Statement

The authors declare that the research was conducted in the absence of any commercial or financial relationships that could be construed as a potential conflict of interest.
